# Combined Stereotactic Body Radiotherapy and Immunotherapy Versus Transarterial Chemoembolization in Locally Advanced Hepatocellular Carcinoma: A Propensity Score Matching Analysis

**DOI:** 10.3389/fonc.2021.798832

**Published:** 2021-12-07

**Authors:** Chi-Leung Chiang, Keith Wan-Hang Chiu, Francis Ann-Shing Lee, Feng-Ming Spring Kong, Albert Chi-Yan Chan

**Affiliations:** ^1^ Department of Clinical Oncology, The University of Hong Kong, Hong Kong, Hong Kong SAR, China; ^2^ Department of Diagnostic Radiology, The University of Hong Kong, Hong Kong, Hong Kong SAR, China; ^3^ Department of Clinical Oncology, Tuen Mun Hospital, Hong Kong, Hong Kong SAR, China; ^4^ Department of Surgery, The University of Hong Kong, Hong Kong, Hong Kong SAR, China

**Keywords:** transarterial chemoembolization, stereotactic body radiotherapy, immunotherapy, hepatocellular carcinoma, immune checkpoint inhibitors, radiotherapy, liver cancer

## Abstract

Immunotherapy has achieved modest clinical activity in HCC patients. Propensity score matching analysis was conducted to compare the efficacy and safety of combined stereotactic SBRT-IO versus TACE in patients with locally advanced HCC in a tertiary center of Hong Kong. Patients with locally advanced HCC who were medically inoperable for, refractory to, or refused to curative surgical interventions were eligible. The primary outcome was PFS; the secondary outcomes were OS, ORR as per mRECIST version 1.1, and TRAEs. Matching pair analysis was performed to compare the clinical outcomes. A total of 226 patients were eligible. Approximately 16 patients in the SBRT-IO group were matched with 48 patients treated with TACE. The median tumor size was 10 cm (range: 2.9–19.6 cm) and 20.3% of the patients had portal vein invasion. The 12- and 24-month PFS were significantly better in the SBRT-IO group (93.3% vs 16.7% and 77.8% vs 2.1%, respectively, p <0.001); the 12- and 24-month OS were also better in the SBRT-IO arm (93.8% vs 31.3% and 80.4% vs 8.3%, respectively, p <0.001). The ORR was 87.5% (CR: 50%, PR: 37.5%) in SBRT-IO arm compared to 16.7% (CR: 2.4%, PR: 14.3%) in those receiving TACE alone (p <0.001). There were fewer ≥grade 3 TRAE (60.4% vs 18.8%, p = 0.004) and treatment discontinuations (25% vs 12.5%, p = 0.295) due to adverse events in the SBRT-IO arm. SBRT-IO had significant superior survival and less treatment toxicity than TACE in patients with locally advanced HCC. Our results provide rationale for studying this combination therapy in prospective randomized trials.

## Introduction

Recent advances in cancer immunotherapy have profoundly influenced the care of patients with hepatocellular carcinoma (HCC). Programmed cell death protein 1/programmed death-ligand 1 (PD-1/PD-L1) targeted therapies have been increasingly used as first-line and second-line treatments of patients with advanced HCC (1–3). However, response rates are modest; overall the response rates to anti PD-1 monotherapy is around 20%, and even combination therapies of atezolizumab/bevacizumab, pembrolizumab/lenvatinib, or nivolumab/ipilimumab are not higher than 50% (4–6). Because the primary resistance of HCC may underlie these low response rates, strategies to overcome these primary or secondary resistances to immune checkpoint inhibitors (ICI) using combination therapies such as combined stereotactic body radiotherapy and immunotherapy (SBRT-IO) are under investigation.

Radiotherapy (RT) has been shown to enhance immunotherapeutic effects. RT can prime the immune system by enhancing antigen presentation, promoting the infiltration of cytotoxic T-cells, and reprogramming the tumor microenvironment against the immune evasion of cancer, while ICI can reverse the RT-mediated exhaustion pathway (7). SBRT-IO has been reported in several cancers, potentially improve the clinical outcome of patients (8, 9). But, evidence on the combination of ICI and SBRT in HCC patients is lacking.

We recently reported encouraging results of SBRT-IO in a small pilot of patients with locally advanced unresectable HCC (10). This study aimed to compare the clinical outcomes of these patients treated with SBRT-IO versus TACE, the current the standard of care in this population.

## Materials and Methods

### Study Design and Population

This was an Institutional Review Board (IRB) approved retrospective study (IRB number: UW-20-674) conducted at a tertiary referral center of Hong Kong. Data was retrieved from a prospectively collected HCC database at the Queen Mary Hospital, Hong Kong. Patients with histological or radiological HCC, who were ineligible for, refractory to, or refused curative surgical interventions, were candidates of loco-regional treatment. All cases were discussed in the multi-disciplinary tumor board. TACE was the standard of care. An experimental treatment of SBRT-IO was offered as an alternative since 2017 based on the potential synergistic effect between SBRT and ICI (10). While the optimal sequence of combining SBRT and ICI remains controversial, we deliver SBRT prior immunotherapy based on the immune-activation property of radiation to sensitize the tumor for subsequent PD-1 inhibitors (11). The advantages and disadvantages of these local treatments were informed to patients and the final treatment depends on patients’ decisions.

The inclusion criteria were as follows: (a) patients were ineligible or refractory to curative surgical interventions; (b) a Child–Pugh (CP) liver score of A5 to B7; (c) tumor nodules ≤5; (d) no main trunk of portal vein invasion (Vp4); (e) no prior systemic therapy, and (f) absence of extra-hepatic metastasis, ascites or encephalopathy. There were no limits on the maximum diameters of tumors.

All patients who received SBRT-IO or TACE from January 2010 to May 2020 were included. Propensity score matching was performed using the nearest neighboring method in 3:1 ratio according to age, sex, tumor size, numbers, and portal vein invasion between the TACE and SBRT-IO groups.

### Transarterial Chemoembolization

TACE was performed by supra-selective cannulation of all the branches supplying the tumor. The emulsion was prepared by mixing lipiodol with cisplatin (1 mg/ml) in a 1:1 ratio using the pumping method, which was then slowly injected under fluoroscopic monitoring according to the size of the tumor and the arterial blood flow. TACE was repeated in eight-week intervals (12).

### Stereotactic Body Radiotherapy and Immunotherapy

For SBRT planning, patients were immobilized *via* a vacuum foam bag (Vac-LokTM; MEDTEC, Iowa, USA) and active breathing control to reduce the amplitude of liver motion. Imaging was performed on the inhale breath-hold contrast computed tomography (CT). GTV was defined as tumor focus that was visualized on contrast imaging. The clinical target volume (CTV) was defined as GTV plus a margin of 0–3 mm. The individualized PTV margins were formulated to compensate the respiratory motion and set-up errors. Cone beam CT was acquired on board before each treatment. The largest tumor was selected as index lesion of SBRT, while maximum three nodules were allowed provided that the liver tolerance dose can be met. The dose was prescribed according to the Radiation Therapy Oncology Group (RTOG) 1112 protocol (23). The total dose of 25 to 50 Gy in five fractions was allowed per institutional protocol. The prescription isodose should encompass 95% of PTV. The final dose was determined such that a maximum tumoricidal dose could be delivered to tumors while respecting the tolerance dose of OAR to the limits of RTOG 1112.

Among our patient cohort, a total dose ranging from 25 to 37.5 Gy in five fractions was given during 1–2 weeks. At 2 weeks after SBRT, intravenous nivolumab at a dose of 3 mg/kg was started and was given every 2 weeks, median 10 cycles (range: 1–20 doses) were given.

### Evaluation of Treatment Response and Decision on Treatment Discontinuation

Contrast computed tomography was performed every 8–12 weeks in the first two years. All radiological responses were evaluated according to the Modified Response Evaluation Criteria for Solid Tumors (mRECIST) version 1.1. Treatment-related adverse events (TRAEs) were graded using the National Cancer Institute Common Terminology for Adverse Events (CTCAE) version 4.0. Treatments were continued until disease progression, unacceptable toxicities, refusal of patients, or achieved radiological complete remission (CR).

### Statistical Analysis

The primary endpoint was progression-free survival (PFS), which was defined as the period from the date of commencement of the treatment to the time of disease progression (either progression of treated lesion, elsewhere in liver, or development of distant metastases), as per mRECIST, or death, whichever occurred earlier. The secondary endpoints included overall survival (OS), objective response rate (ORR), TRAEs, and liver function deterioration. OS was defined as the period from the date of commencement of the study treatment to the date of death or last follow-up, whichever occurred earlier. Radiological response was recorded per lesion according to the mRECIST. Disease control rate (DCR) was defined as percentage of patient attained radiological complete response (CR), partial response (PR) or stable disease for ≥6 months. Liver function deterioration was defined as progression of Child–Pugh score of ≥2.

Continuous variables were presented as medians and ranges. Comparison between the groups was carried out using the Chi-squared or Mann–Whitney U test where appropriate. Survivals were studied with the Kaplan–Meier method. Cox proportional hazard regression model was used to determine independent prognostic factors. Statistical significance was defined as p <0.05, and all the performed tests were two-tailed. Data was analyzed using R version 3.25 (Vienna, Austria).

## Results

### Patients and Treatments

A total of 226 patients with HCC were eligible and enrolled in the present study, namely, 210 patients who initially received TACE and the remaining 16 who were treated with SBRT-IO. **Table 1** shows the baseline and tumor characteristics of all the patients and their significances for clinical outcome.

**Table 1:** Baseline demographics and tumour characteristics of all patients.

	Before propensity score matching			After propensity score matching	
	Unmatched TACE N = 210	SBRT-IO N = 16	P-value	Matched TACE N = 48	P-value
**Age (median, range) years**	69 (36-94)	66.5 (38–86)	0.504	73 (49–87)	0.149
**Sex (n, % male)**	158 (75.2)	14 (87.5)	0.268	43 (89.6)	0.817
**Hepatitis B carrier (n, %)**	129 (61.4)	12 (75.0)	0.280	26 (54.2)	0.142
**ECOG 0–1 (n, %)**	192 (91.4)	12 (75.0)	0.439	45 (93.8)	0.407
**Child–Pugh class A (n, %)**	182 (86.7)	14 (87.5)	0.925	46 (95.8)	0.233
**ALBI grade**			0.531		0.238
** 1**	76 (36.2)	8 (50.0)		15 (31.2)	
** 2**	121 (57.6)	7 (43.8)		32 (66.7)	
** 3**	13 (6.2)	1 (6.2)		1 (2.1)	
**Albumin (g/L)**	37 (17–48)	39 (30–45)	0.250	37 (25–45)	0.192
**Bilirubin (µmol/L)**	13 (4–55)	15 (8–122)	0.171	12.5 (4–39)	0.149
**Platelet (×10^9^/L)**	169.5 (25–551)	234 (79–402)	0.069	226 (66–522)	0.773
**INR**	1.1 (0.8–2.3)	1.1 (1–1.5)	0.339	1.1 (0.9–1.6)	0.543
**BCLC stage (n, %)**			0.002		0.998
** A**	79 (37.6)	3 (18.8)		9 (18.7)	
** B**	99 (47.2)	5 (33.3)		15 (31.3)	
** C**	32 (15.2)	8 (50)		24 (50)	
**Tumor number (n, %)**			0.518		0.460
** 1**	89 (42.4)	9 (56.2)		27 (56.3)	
** 2**	26 (12.4)	2 (12.5)		1 (2.1)	
** ≥3**	95 (45.2)	5 (31.3)		20 (41.6)	
Tumor size (cm)*	6.95 (1–19.6)	10 (3.4–18)	0.016	10.4(2.68–19.6)	1.000
**Portal vein invasion (n, %)**	19 (9.1)	3 (18.8)	0.001	10 (20.8)	0.827
**AFP ≥ 200 ng/ml (n, %)**	84 (40.0)	7 (43.8)	0.768	21 (43.8)	1.000
**Range**	1–1,458,960	3–499,988		2–362,901	

TACE, transarterial chemoembolization; SBRT-IO, combined stereotactic body radiotherapy and immunotherapy; ECOG, Eastern Cooperative Oncology Group; INR, international normalized ratio; BCLC, Barcelona Clinic Liver Cancer; AFP, alpha-feto protein.

^*^Tumor size of the largest lesion.

The SBRT-IO group had higher percentage of patients with Barcelona Clinic Liver Cancer (BCLC) stage C disease and portal vein invasion; median size of tumor was larger in the SBRT-IO group (10 cm vs. 6.95 cm, p = 0.016). After propensity score matching, a total of 48 patients treated with TACE were identified to match the 16 patients treated with SBRT-IO. No significant difference was observed between-group. Overall, around 90% of analyzed patients were male and had a performance status of ECOG 0–1, and 60% were hepatitis B carrier.

### Patients Population Between TACE and SBRT-IO

Among the 64 included patients after matching, the median size of tumor was 10 cm (range: 3.4–19.6 cm) and 20.3% of the patients had portal vein invasion. Among the 48 patients matched in the TACE arm, median 2 sessions of TACE (range: 1–16) were given. For the SBRT-IO arm, a median dose of 35 Gy (range: 27.5–37.5 Gy) was prescribed and median 10 cycles of nivolumab (range: 1–20 doses) were given. Total 24 lesions were irradiated in SBRT-IO arm. (N = 11, single lesion; N = 2, two lesions; N = 3, three lesions). One and six patients received post-progression therapies in the SBRT-IO and TACE arms, respectively. Neither patient in the TACE arm received SBRT or immunotherapy, nor patient in SBRT-IO arm received TACE after progression (refer to **Table S1** for detailed information).

### Overall Survival and Progression-Free Survival Between TACE and SBRT-IO

**Figure 1:**
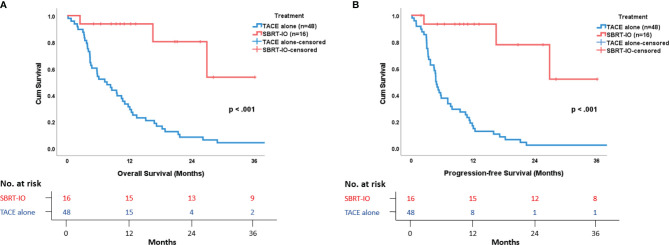
Survival Outcome in Patients with Locally Advanced Unresectable Hepatocellular Carcinoma. **(A)** Overall survival and **(B)** Progression-free survival are remarkably and significantly better in SBRT-IO group versus matched TACE group.

The survival data was censored on December 31, 2020. The median follow-up time of the SBRT-IO and matched TACE groups were 12.7 months (range: 2.5–36.1 months) and 7.4 months (range: 0.2–57.2 months), respectively. The 6-, 12-, and 24-month PFS were better in the SBRT-IO group (93.3% vs. 37.5%, 93.3% vs. 16.7%, and 77.8% vs. 2.1%, respectively, p <0.001). The median PFS of the SBRT-IO group was not reached (range: 1.9–36.1 months) compared to 4.83 months (range: 0.2–42.2 months) of the TACE group. The 6-, 12-, and 24-month OS were also better in the SBRT-IO group (93.8% vs. 54.2%, 93.8% vs. 31.3%, and 80.4% vs. 8.3%, respectively, p <0.001). The median OS of the SBRT-IO group was not reached (range: 2.5–36.1 months) compared to 7.44 months (range: 0.2–57.2 months) of the TACE group (as shown in **Figure 1**).

At the time of analysis, there were three deaths out of 16 patients in the SBRT-IO arm, and all the 48 patients died in the matched TACE group. All of the three patients who died in the SBRT-IO group showed no evidence of disease progression; two died of community-acquired pneumonia and one died of hemobilia. In the matched TACE group, 37 deaths (77.1%) were cancer related, four died of pulmonary causes, two died of intra-cranial hemorrhage, and one died of myocardial infarction, liver abscess, liver decompensation, intestinal obstruction, or unknown cause (each). Among patients in the matched TACE group, intra-hepatic progression (90.6%) represented the dominant mode of failure; there were no significant differences in the PFS or OS between the different treatment periods from 2010 to 2020 (as shown in **Figure S1**).

Under the multi-variable analysis, SBRT-IO, was an independent predictor of better OS (hazard ratio (HR) = 0.14, range: 0.30–0.96, p = 0.036) and better PFS (HR = 0.1, range: 0.03–0.33, p <0.001). Tumor number was another independent predictor of OS (HR = 0.54, range: 0.30–0.96, p = 0.036) and PFS (HR = 0.38, range 0.21–0.72, p = 0.003) (**Table 2**).

**Table 2:** Univariate and multivariate analyses of potential prognostic factors affecting overall and progression-free survival after propensity score matching.

For matched groups (n = 64):	Overall Survival						Progression-free Survival					
	UVA			MVA			UVA			MVA		
	HR	95% CI	P	HR	95% CI	P	HR	95% CI	P	HR	95% CI	P
**SBRT-IO vs. TACE**	0.13	0.04–0.42	<0.001	0.14	0.04–0.46	0.001	0.10	0.03–0.32	<0.001	0.10	0.03–0.33	<0.001
**Age (<60 vs. ≥60 years)**	1.24	0.58–2.67	0.58				1.37	0.64–2.94	0.42			
**Sex (male vs. female)**	1.25	0.49–3.16	0.64				1.50	0.59–3.81	0.39			
**Hepatitis B carrier (yes vs. no)**	1.03	0.59–1.81	0.92				0.99	0.56–1.73	0.97			
**ECOG (0–1 vs. 2)**	1.92	0.75–4.90	0.17				2.27	0.89–5.81	0.09			
**Child–Pugh class (A vs. B)**	2.08	0.50–8.61	0.31				1.91	0.46–7.92	0.37			
**ALBI grade (1 vs. 2)**	0.63	0.34-1.15	0.13				0.52	0.28-0.96	0.04	0.90	0.49–1.66	0.73
**Portal vein invasion (Yes vs. no)**	1.08	0.55–2.12	0.82				1.13	0.51–2.50	0.76			
**BCLC stage (A vs. B)**	1.15	0.50–2.64	0.74				1.19	0.46–3.08	0.72			
**BCLC stage (A vs. C)**	0.77	0.36–1.68	0.52				0.77	0.31–1.87	0.56			
**Tumor number (n = 1–2 vs. ≥3)**	0.45	0.25–0.80	0.007	0.54	0.30–0.96	0.036	0.38	0.21–0.70	0.002	0.38	0.21–0.72	0.003
**Tumor size (<10 cm vs. ≥10 cm)**	0.60	0.34–1.08	0.09				0.59	0.33–1.05	0.07			
**AFP (<200 vs. ≥200 ng/ml)**	0.71	0.40–1.25	0.24				0.67	0.38–1.19	0.17			

TACE, transarterial chemoembolization; SBRT-IO, combined stereotactic body radiotherapy and immunotherapy; ECOG, Eastern Cooperative Oncology Group; INR, international normalized ratio; BCLC, Barcelona Clinic Liver Cancer; AFP, alpha-feto protein; UVA, univariate analysis; MVA, multivariate analysis; HR, hazard ratio; CI, confidence. interval.

### Overall Survival and Progression-Free Survival Between TACE and SBRT-IO

**Figure 2:**
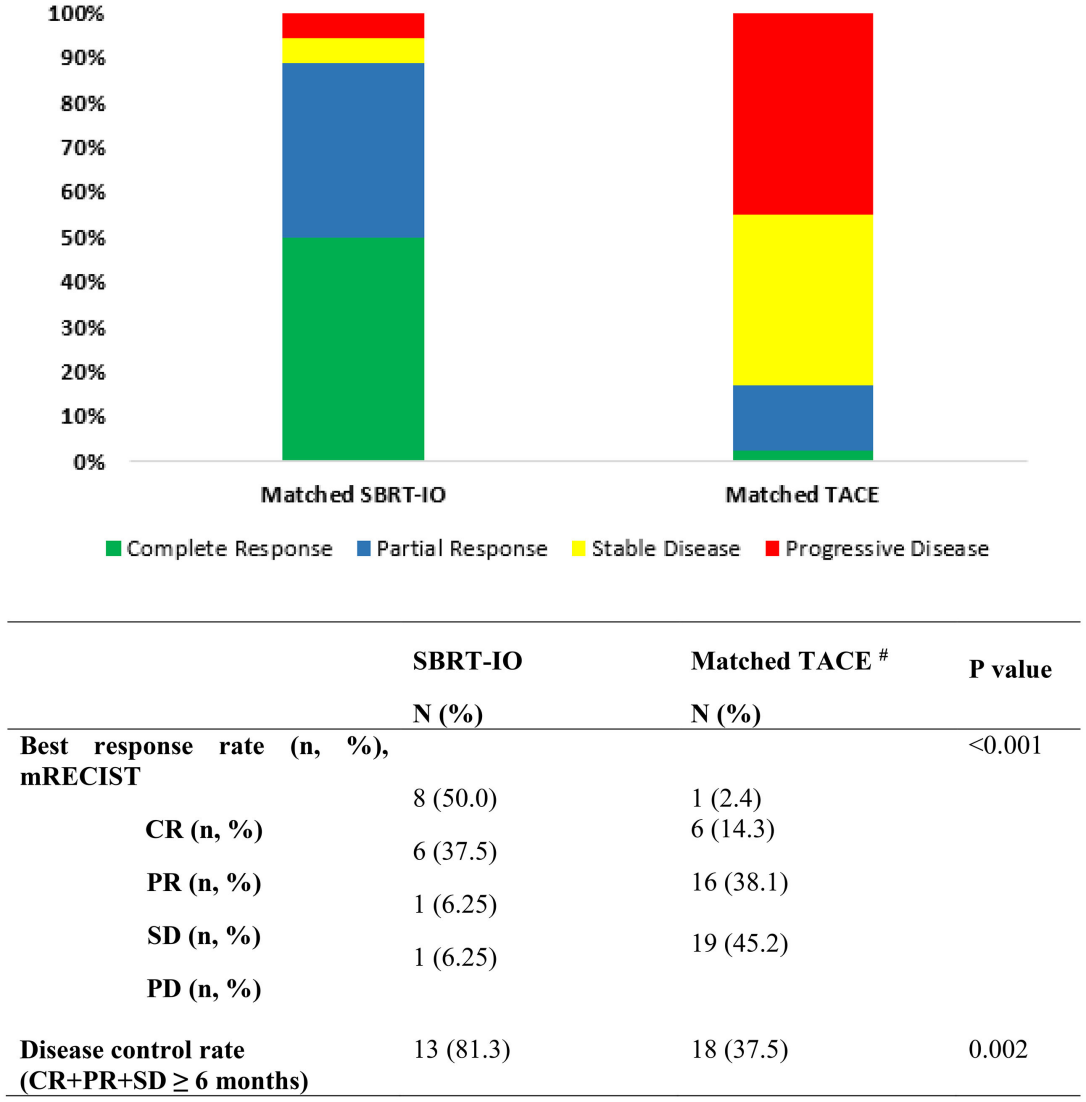
The best mRECIST of the matched TACE and SBRT-IO patients. SBRT-IO, combined stereotactic body radiotherapy and immunotherapy; TACE, transarterial chemoembolisation; mRECIST, modified response evaluation criteria in solid tumours; CR, complete response; PR, partial response; SD, stable disease; PD, progressive disease; N, number of lesions. #6 subjects in the matched TACE cohort did not have follow-up scan for tumour reassessment.

**Figure 3:**
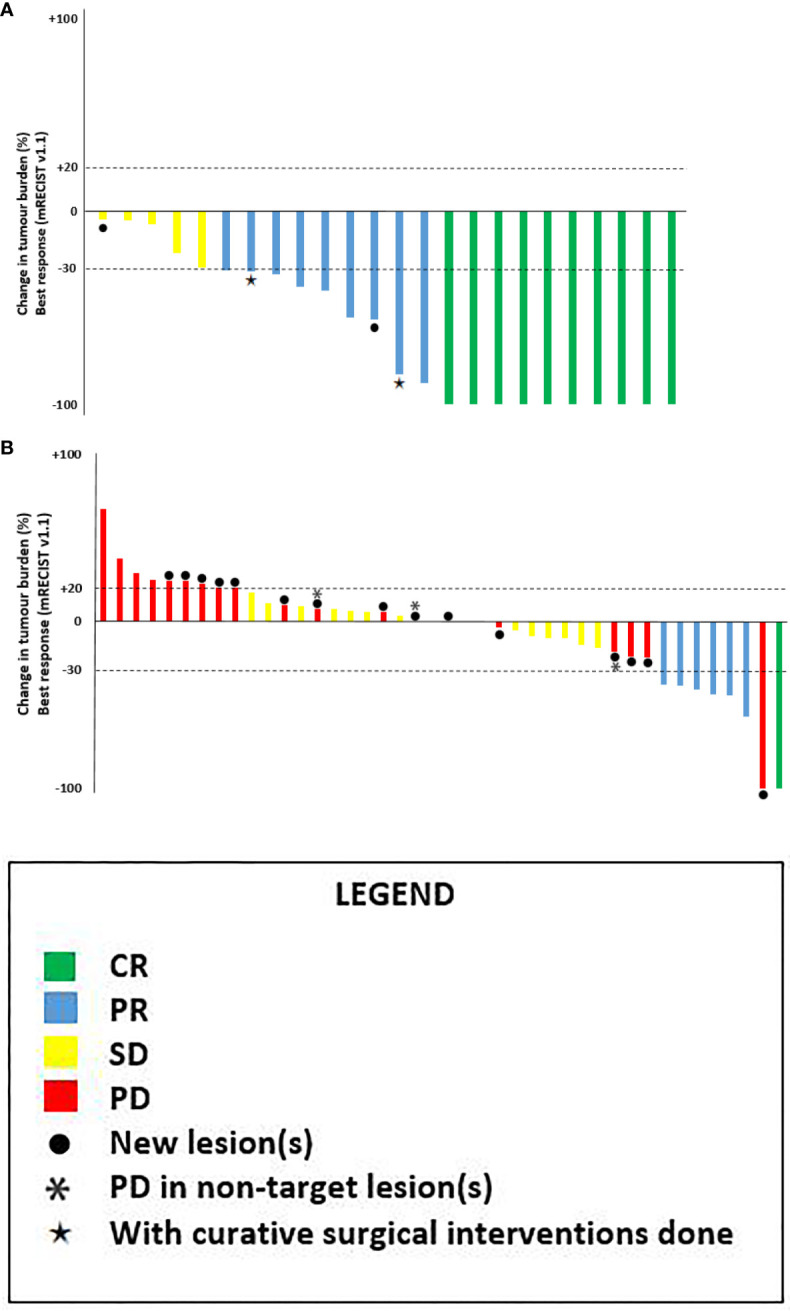
Waterfall plots of best overall response of the target lesion(s) as per mRECIST v1.1. **(A)** SBRT-IO arm: 16 patients with total 24 lesions*. **(B)** Matched-TACE arm: 42 lesions in 48 matched patients (6 of them didn’t have tumour reassessment). CR, complete response; PR, partial responses; SD, stable disease; PD, progressive disease. ●: patients exhibiting new lesions at subsequent evaluation, *: progression in non-target lesions, ★: patients with curative surgical interventions done after responding to initial treatment.

**Figure 2** depicts the best objective response of 16 patients in SBRT-IO arm and 42 evaluable patients in matched-TACE arm. Six patients of TACE arm did not have reassessment scan due to rapid deterioration. The ORR was significantly higher in the SBRT-IO group (87.5% vs. 16.7%, p <0.001). DCR was also significantly better in the SBRT-IO group (81.3% vs. 37.5%, p = 0.002). In the matched TACE group, 24 patients (50%) never had radiological disease controlled. The off-target progression represented the dominant mode of treatment failure in TACE arm, which occurred in 15 of 42 evaluable patients (35.7%). In contrast, only one patient (6.3%) developed progressive disease after SBRT-IO; this patient developed a new HCC focus outside the irradiated field and two SBRT-treated lesions had partial response (PR) and static disease (SD). Two patients of the SBRT-IO arm (12.5%) had radiofrequency ablation (RFA) performed after PR, with complete clearance of the tumour subsequently achieved. The waterfall plot of **Figure 3** illustrates the treatment response of index lesion in the SBRT-IO and TACE group.

Of note, Nivolumab was stopped for eight patients who achieved CR after median 7.1 months of treatment (range: 2.1–15.6 months); none of them developed relapse in the median follow-up time of 5.7 months (range: 0.7–25.0 months).

### Treatment Related Adverse Events and Liver Function Deterioration Between TACE and SBRT-IO

Risk of ≥grade 3 TRAEs and discontinuation of treatment due to toxicities were more common in patients who received TACE (60.4% vs. 18.8%, p = 0.004; 25% vs. 12.5%, respectively, p = 0.295). There was more elevated transaminase, anemia, leukopenia, and fever in the TACE group, while patients who received SBRT-IO had more fatigue, diarrhoea, and rash. Among patients treated with SBRT-IO, none developed classical radiation induced liver disease, and there were no treatment-related deaths reported. There were fewer patients who developed Child–Pugh score progression ≥2 at 3 months (6.7% vs. 20.9%, p = 0.008), 6 months (6.7% vs. 12.0%, p = 0.021), and 12 months (0% vs. 21.4%, p <0.001) in the SBRT-IO arm compared to the TACE arm (**Table 3**).

**Table 3:** Treatment related adverse event and Child-Pugh score progression of SBRT-IO vs. matched TACE.

	SBRT-IO (N = 16)		Matched TACE (N = 48)		P-value
	Any Grade	Grades 3–4	Any Grade	Grades 3–4	
	Number (%)		Number (%)		
**Treatment-related AEs**		3 (18.8%)		29 (60.4%)	0.004
**AEs lead to discontinuation**	2 (12.5%)	1 (6.3%)	12 (25%)	5 (10.4%)	0.295
**Treatment-related death**	0 (0%)	0 (0%)	2 (4.2%)	1 (2.1%)	0.407
					
**Hemoglobin**	8 (50%)	0 (0%)	38 (79.2%)	4 (8.3%)	0.06
**Leukocytes**	2 (12.5%)	0 (0%)	24 (50%)	0 (0%)	0.025
**Platelet**	12 (75%)	1 (6.3%)	26 (54.2%)	6 (12.5%)	0.251
					
**Bilirubin**	5 (31.3%)	1 (6.3%)	20 (41.7%)	5 (10.4%)	0.617
**AST/ALT**	15 (93.7%)	0 (0%)	43 (89.6%)	25 (52.1%)	<0.001
**Nausea and vomiting**	4 (25%)	0 (0%)	13 (27.1%)	0 (0%)	0.456
**Diarrhea**	3 (18.8%)	0 (0%)	1 (2%)	0 (%)	0.002
**Appetite lost**	1 (6.3%)	0 (0%)	9 (18.9%)	0 (0%)	0.477
					
**Fatigue**	10 (62.5%)	0 (0%)	9 (18.7%)	2 (4.2%)	0.003
**Fever**	3 (18.8%)	0 (0%)	23 (47.9%)	0 (0%)	0.04
**Weight loss**	1 (6.3%)	0 (0%)	8 (16.7%)	0 (0%)	0.562
**Pain**	3 (18.8%)	0 (0%)	24 (50%)	0 (0%)	0.028
**Rash**	5 (31.3%)	0 (0%)	4 (8.3%)	0 (0%)	0.002
**Pruritus**	2 (12.5%)	1 (6.3%)	4 (8.3%)	0 (0%)	0.213
**Adrenal insufficiency**	1 (6.3%)	0 (0%)	1 (2.1%)	0 (0%)	0.407
					
**Progression of CP score ≥2**					
**3 months**	1/15 (6.7%)		9/43 (20.9%)		0.008
**6 months**	1/15 (6.7%)		3/25 (12.0%)		0.021
**12 months**	0/8 (0%)		3/14 (21.4%)		<0.001

TACE, transarterial chemoembolization; SBRT-IO, combined stereotactic body radiotherapy and immunotherapy; AEs, adverse events; AST, Aspartate transaminase; ALT, Alanine transaminase; CP, Child–Pugh.

The incidence of only toxicities ≥5% is shown.

## Discussion/Conclusion

To our knowledge, this is the first comparative study to evaluate the combined SBRT-IO in HCC population. Our findings clearly demonstrated the promising anti-tumor activity of combined SBRT-IO among patients with locally advanced HCC. This present analysis reported that patients who received SBRT-IO had statistically significant better PFS, OS, and ORR than those who received TACE. Around 90% of the patients treated with SBRT-IO survived without disease progression at 1 year and 50% had achieved CR. The high ORR of 88.8% was also superior over that of ICI reported in previous studies (1–6).

Although TACE is widely used in patients with unresectable HCC, the prognoses vary as patients selected for TACE is highly heterogeneous in tumor burden, liver function, and treatment histories. Recent studies suggested patients with tumor burden beyond the up-to-seven criteria are unlikely to respond to TACE and their hepatic reserve tends to deteriorate after treatment (13, 14). Recently, Wang et al. showed that patients who were early refractory to TACE had significantly worse prognoses (PFS: 4.36 months vs. 12.2 months) than those responded to treatment (15). Similarly, our patient cohort had extensive tumor burden and most became refractory to TACE after median of two sessions; hence, the prognosis of matched-TACE arm was poor with median PFS 4.83 months, which is similar with the literature (15). Our study had provided a novel therapeutic approach for those who respond poorly to TACE.

There were several reasons accounted for the promising activity of SBRT-IO combination. First, previous studies had demonstrated that SBRT achieved excellent local control (1-year local control rate of 77–87%) in locally advanced HCC, but the competing risk of metastasis had resulted in later precipitous drop of OS (median OS of 9–17 months) (16, 17). Notably, only one patient (6.3%) treated with SBRT-IO in our study developed out-of-field failure compared to that of 35.7% (15 out of 42 evaluable patients) in the TACE arm. We postulated that the immune-modulatory effect of SBRT has augmented the effect of ICI in eradicating the occult metastasis; this phenomenon, known as ‘systemic therapy augmented by radiotherapy (STAR)’, has also been reported in NSCLC (18). Second, because of the extensive tumor load in our patient cohort, we were only allowed to prescribe non-ablative dose of radiation (5.5–7.5 Gy × 5) to respect the radiation tolerance of liver (16, 17). Nevertheless, excellent local tumor response (ORR: 87.5%, CR rate: 50%) was achieved. A study by Vanpouille-Box et al. provided an important mechanistic clue regarding the modulation of the immunogenic effect by different radiation dose/fractionation schemes. They showed that modest dose radiation (8 Gy × 3) achieved similar local control as single ablative dose (30 Gy) in the concurrent use of ICI, but better systemic responses are achieved with an increased IFN-β production *via* the cGAS/STING pathway. Our findings are consistent with the pre-clinical data suggesting that ICI can lower the radiation dose required to induce the same tumor response (19). Lastly, we stopped the anti-PD-1 therapy in eight patients once they attained radiological CR; the decision was made at the discretion of clinicians in agreement with the patients. Interestingly, none of them relapsed after discontinuation of therapy for up to 2 years. The optimal duration of ICI remains unknown; yet a previous study has also suggested that the risk of progression or death is low among patients who achieved radiological CR (20). Longer follow-up is needed in our cohort to ascertain the durability of response; nevertheless, the encouraging CR rate and the durable response experienced with SBRT-IO provide hope for a cure for unresectable HCC patients without the need for additional therapy, a goal that previously seemed unachievable.

There was no abnormal safety signal observed in combined SBRT and ICI. Patients treated with SBRT-IO had better safety profile and tolerance. More importantly, treatment of TACE often leads to the deterioration of liver function that robs the patients of the opportunity of subsequent systemic therapy (13, 14). Our data suggested that SBRT-IO might better preserve the liver function of patients compared with TACE.

Our data supported the benefit of SBRT-IO in HCC patients. Prospective studies are required to validate our findings in a broader population and compare its efficacy with SBRT alone or ICI alone; future studies should also be prioritized to define the optimal timing, dosing, and treatment volume of radiotherapy and the role of PD-L1 status. Correlative studies are needed to define the mechanistic rationale behind the synergy of SBRT-IO. Lastly, recent data suggested TACE may also favorably modulate the tumor microenvironment and potentially can effectively combine with ICI; a number of trials are now on-going to evaluate the synergy of this combination (21); comparative study of SBRT-IO vs. TACE-IO is warranted when more data become available in the future.

There were several limitations of this study. First, it was a retrospective and single-center study with a small sample size; therefore, the selection bias could not be entirely eliminated. However, we had followed the patients in both arms under a unified protocol with regular imaging schedule so that the biases of PFS assessment were minimized, thus the reliability of the superior results of OS and ORR of SBRT-IO. Second, the relatively short duration of follow-up rendered the assessment of late toxicity and long-term survival not feasible. Also, the difference in the treatment period may introduce bias in favor of the survival outcome in the SBRT-IO arm, in which majority of the patients were treated in recent years with expanding therapeutic options. Nonetheless, our findings clearly demonstrated the superiority of SBRT-IO over TACE in terms of PFS and tumor response; additionally, the survival of the TACE arm has been fairly consistent over the years. Finally, the PD-L1 status and its impact on the treatment outcome were not evaluated in the present study; however, the high ORR of SBRT-IO suggested the combination worked regardless of the PD-L1 status.

In conclusion, our findings provide rationale to study the SBRT-IO treatment among locally advanced HCC patients in prospective randomized studies.

## Data Availability Statement

The original contributions presented in the study are included in the article/**Supplementary Material**. Further inquiries can be directed to the corresponding authors.

## Ethics Statement

This study was approved by The University of Hong Kong/Hospital Authority Hong Kong West Cluster Institutional Review Board (IRB number: UW-20-674). The patients/participants provided their written informed consent to participate in this study. Written informed consent was not obtained from the individual(s) for the publication of any potentially identifiable images or data included in this article.

## Author Contributions

Conception and design: C-LC, AC, and KC. Collection and assembly of data: C-LC and AC. Data analysis and interpretation: C-LC and AC. Manuscript writing: All authors. All authors contributed to the article and approved the submitted version.

## Conflict of Interest

C-LC reports receiving research funding of AstraZeneca, Merck Kgga, and Taiho. He had a consulting or advisory role at AstraZeneca and Eiasi.

The remaining authors declare that the research was conducted in the absence of any commercial or financial relationships that could be construed as a potential conflict of interest.

## Publisher’s Note

All claims expressed in this article are solely those of the authors and do not necessarily represent those of their affiliated organizations, or those of the publisher, the editors and the reviewers. Any product that may be evaluated in this article, or claim that may be made by its manufacturer, is not guaranteed or endorsed by the publisher.

## Abbreviations

CR, Complete remission; DCR, Disease control rate; ECOG, Eastern Cooperative Oncology Group; HCC, Hepatocellular carcinoma; HR, Hazard ratio; ICI, Immune checkpoint Inhibitor; IRB, Institutional review board; mRECIST, Modified Response Evaluation Criteria in Solid Tumors; ORR, Objective response rate; OS, Overall survival; PD-1, Program cell death protein 1; PD-L1, Programmed death-ligand 1; PFS, Progression-free survival; PR, Partial remission; RTOG, Radiation Therapy Oncology Group; SBRT-IO, Combined stereotactic body radiotherapy and immunotherapy; TACE, Transarterial chemoembolization; TRAE, Treatment-related adverse event.

## References

[1] El-Khoueiry AB Sangro B Yau T Crocenzi TS Kudo M Hsu C. Nivolumab in Patients With Advanced Hepatocellular Carcinoma (CheckMate 040): An Open-Label, Non-Comparative, Phase 1/2 Dose Escalation and Expansion Trial. Lancet (2017) 389:2492–502. doi: 10.1016/S0140-6736(17)31046-2 PMC7539326 28434648

[2] Zhu AX Finn RS Edeline J Cattan S Ogasawara S Palmer D. Pembrolizumab in Patients With Advanced Hepatocellular Carcinoma Previously Treated With Sorafenib (KEYNOTE-224): A non-Randomised, Open-Label Phase 2 Trial. Lancet Oncol (2018) 19:940–52. doi: 10.1016/S1470-2045(18)30351-6 29875066

[3] Yau T Park JW Finn RS Cheng A-L Mathurin P Edeline J. CheckMate 459: A Randomized, Multi-Center Phase III Study of Nivolumab (NIVO) vs Sorafenib (SOR) as First-Line (1L) Treatment in Patients (PTS) With Advanced Hepatocellular Carcinoma (aHCC). Ann Oncol (2019) 30:v874–5. doi: 10.1093/annonc/mdz394.029

[4] Finn RS Qin S Ikeda M Galle PR Ducreux M Kim T-Y. Atezolizumab Plus Bevacizumab in Unresectable Hepatocellular Carcinoma. N Engl J Med (2020) 382:1894–905. doi: 10.1056/NEJMoa1915745 32402160

[5] Yau T Kang Y-K Kim T-Y El-Khoueiry AB Santoro A Sangro B. Efficacy and Safety of Nivolumab Plus Ipilimumab in Patients With Advanced Hepatocellular Carcinoma Previously Treated With Sorafenib: The CheckMate 040 Randomized Clinical Trial. JAMA Oncol (2020) 6(11):e204564. doi: 10.1001/jamaoncol.2020.4564 33001135 PMC7530824

[6] Finn RS Ikeda M Zhu AX Sung MW Baron AD Kudo M. Phase Ib Study of Lenvatinib Plus Pembrolizumab in Patients With Unresectable Hepatocellular Carcinoma. J Clin Oncol (2020) 38(26):2960–70. doi: 10.1200/JCO.20.00808 PMC7479760 32716739

[7] Bernstein MB Krishnan S Hodge JW Chang JY. Immunotherapy and Stereotactic Ablative Radiotherapy (ISABR): A Curative Approach? Nat Rev Clin Oncol (2016) 13:516–24. doi: 10.1038/nrclinonc.2016.30 PMC6053911 26951040

[8] Theelen WSME Peulen HMU Lalezari F van der Noort V de Vries JF Aerts JGJV. Effect of Pembrolizumab After Stereotactic Body Radiotherapy Versus Pembrolizumab Alone on Tumor Response in Patients With Advanced Non-Small Cell Lung Cancer: Results of the PEMBRO-RT Phase 2 Randomized Clinical Trial. JAMA Oncol (2019) 5:1276–82. doi: 10.1001/jamaoncol.2019.1478 PMC6624814 31294749

[9] Bauml JM Mick R Ciunci C Aggarwal C Davis C Evans T. Pembrolizumab After Completion of Locally Ablative Therapy for Oligometastatic Non-Small Cell Lung Cancer: A Phase 2 Trial. JAMA Oncol (2019) 5:1283–90. doi: 10.1001/jamaoncol.2019.1449 PMC6624820 31294762

[10] Chiang CL Chan ACY Chiu KWH Kong F-MS. Combined Stereotactic Body Radiotherapy and Checkpoint Inhibitor in Unresectable Hepatocellular Carcinoma: A Synergistic Treatment Strategy. Front Oncol (2019) 9:1157. doi: 10.3389/fonc.2019.01157 31799176 PMC6874138

[11] Sharabi AB Lim M DeWeese TL Drake CG. Radiation and Checkpoint Blockade Immunotherapy: Radiosensitisation and Potential Mechanisms of Synergy. Lancet Oncol (2015) 16(13):e498–509. doi: 10.1016/S1470-2045(15)00007-8 26433823

[12] Lo C Ngan H Tso W Liu C Lam C-M Poon RT-P. Randomized Controlled Trial of Transarterial Lipiodol Chemoembolization for Unresectable Hepatocellular Carcinoma. Hepatology (2002) 35(5):1164–71. doi: 10.1053/jhep.2002.33156 11981766

[13] Yasui Y Tsuchiya K Kurosaki M Takeguchi T Takeguchi Y Okada M. Up-To-Seven Criteria as a Useful Predictor for Tumor Downstaging to Within Milan Criteria and Child-Pugh Grade Deterioration After Initial Conventional Transarterial Chemoembolization. Hepatol Res (2018) 48:442–50. doi: 10.1111/hepr.13048 29278654

[14] Arizumi T Minami T Chishina H Kono M Takita M Yada N. Time to Transcatheter Arterial Chemoembolization Refractoriness in Patients With Hepatocellular Carcinoma in Kinki Criteria Stages B1 and B2. Dig Dis (2017) 35:589–97. doi: 10.1159/000480208 29040992

[15] Wang TC An TZ Li JX Zhang Z-S Xiao Y-D. Development and Validation of a Predictive Model for Early Refractoriness of Transarterial Chemoembolization in Patients With Hepatocellular Carcinoma. Front Mol Biosci (2021) 8:633590. doi: 10.3389/fmolb.2021.633590 33816555 PMC8012485

[16] Bujold A Massey C Kim J Brierley J Cho C Wong RKS. Sequential Phase I and II Trials of Stereotactic Body Radiotherapy for Locally Advanced Hepatocellular Carcinoma. J Clin Oncol (2013) 31:1631–9. doi: 10.1200/JCO.2012.44.1659 23547075

[17] Gkika E Schultheiss M Bettinger D Maruschke L Neeff HP Schulenburg M. Excellent Local Control and Tolerance Profile After Stereotactic Body Radiotherapy of Advanced Hepatocellular Carcinoma. Radiat Oncol (2017) 12:116. doi: 10.1186/s13014-017-0851-7 28701219 PMC5508695

[18] Torok JA Samama JK. Combining Immunotherapy and Radiotherapy for the STAR Treatment. Nat Rev Clin Oncol (2019) 16:666–7. doi: 10.1038/s41571-019-0277-2 31541199

[19] Vanpouille-Box C Alard A Aryankalayil MJ Sarfraz Y Diamond JM Schneider RJ. DNA Exonuclease Trex1 Regulates Radiotherapy-Induced Tumour Immunogenicity. Nat Commun (2017) 8:15618. doi: 10.1038/ncomms15618 28598415 PMC5472757

[20] Jansen YJL Rozeman EA Mason R Goldinger SM Geukes Foppen MH Hoejberg L. Discontinuation of Anti-PD-1 Antibody Therapy in the Absence of Disease Progression or Treatment Limiting Toxicity: Clinical Outcomes in Advanced Melanoma. Ann Oncol (2019) 30:1154–61. doi: 10.1093/annonc/mdz110 30923820

[21] Greten TF Mauda-Havakuk M Heinrich B Korangy F Wood BJ. Combined Locoregional-Immunotherapy for Liver Cancer. J Hepatol (2019) 70(5):999–1007. doi: 10.1016/j.jhep.2019.01.027 30738077 PMC6462230

