# Supplementary material for: Combined Stereotactic Body Radiotherapy and Immunotherapy Versus Transarterial Chemoembolization in Locally Advanced Hepatocellular Carcinoma: A Propensity Score Matching Analysis
Source: Front Oncol. 2021 Dec 7;11:798832. doi: 10.3389/fonc.2021.798832 (PMC8688536; doi:10.3389/fonc.2021.798832)
Supplement: Supplementary file 1 [file DataSheet_1.docx]

Supplementary Material

# Supplementary Table

## Supplementary Table 1

**Table S1.** Details of study treatments and post-progression therapies

|  | SBRT-IO  N=16 | Matched TACE  N=48 |
| --- | --- | --- |
| Median sessions of TACE, range | NA | 2 (1–16) |
| Median number of cycles of nivolumab, range | 10 (1 – 20) | NA |
| SBRT dose  Median, range  27.5 Gy in 5 fractions, n  30 Gy in 5 fractions, n  32.5 Gy in 5 fractions, n  35 Gy in 5 fractions, n*  37.5 Gy in 5 fractions, n* | 35 Gy (27.5–37.5 Gy)  1  1  2  7  5 | NA |
| Post-progression therapy, n  Multiple lines of systemic therapies  Sorafenib  SIRT | 1  1  0  0 | 6  0  5  1 |

**Abbreviations:** SBRT-IO, combined stereotactic body radiotherapy and immunotherapy; TACE, transarterial chemoembolisation; SIRT, selective internal radiotherapy; NA, not applicable.

***** One patient with two lesions and one patient with three lesions received 35Gy in 5 fractions. One patient with two lesions and two patients with three lesions received the same SBRT dosage (37.5Gy in 5 fractions) (i.e. total 7 patients with 10 lesions received 35Gy in 5 fractions; total 5 patients with 10 lesions received 37.5Gy in 5 fractions).

# Supplementary Figures

## Supplementary Figure 1

**Figure S1.** Kaplan-Meier curves of (A) PFS and (B) OS in patients of the TACE arm of different time periods

Fig. S1A. Kaplan-Meier curves of PFS


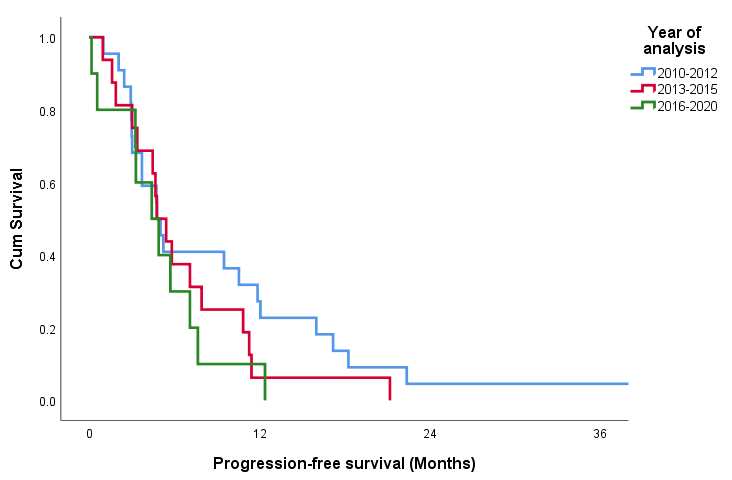


Fig. S1B. Kaplan-Meier curves of OS


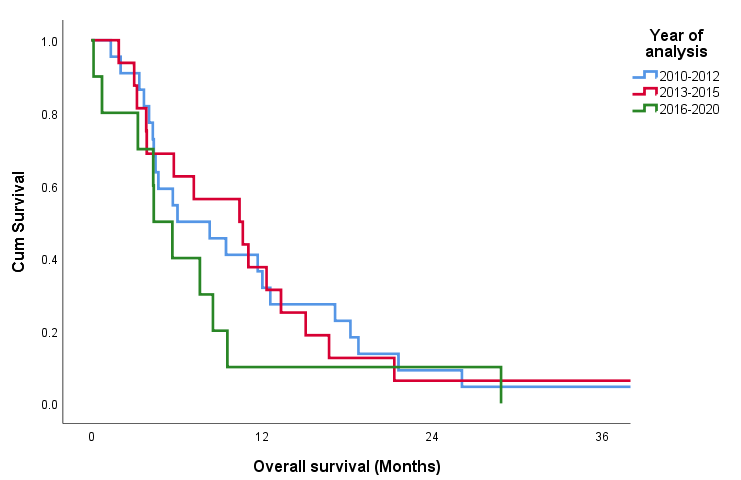


| Time period | N | PFS (months)  Median, range | P value | OS (months)  Median, range | P value |
| --- | --- | --- | --- | --- | --- |
| 2010–2012 | 22 | 5.03 (0.99–42.18) | 0.314 | 7.23 (1.38–57.23) | 0.448 |
| 2013–2015 | 16 | 4.73 (0.95–21.19) |  | 7.23 (1.94–51.02) |  |
| 2016–2020 | 10 | 4.65 (0.16–12.39) |  | 5.06 (0.16–28.88) |  |

**Abbreviations:** TACE, transarterial chemoembolisation; N, number of patients; PFS, progression-free survival; OS, overall survival

## Supplementary Figure 2

**Figure S2.** 69 years old female presented with bi-lobed HCC. The largest lesion (12cm) was irradiated with SBRT (32.5Gy in 5 fractions) followed by 10 cycles of immune checkpoint inhibitors, achieved complete response in both the irradiated lesion and out-of-field lesions.

| **A1** | **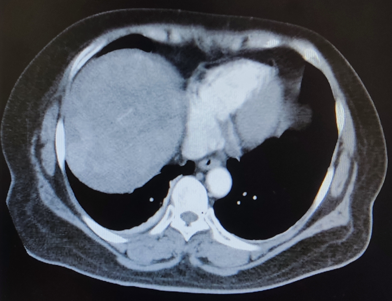** | | | **B1** | **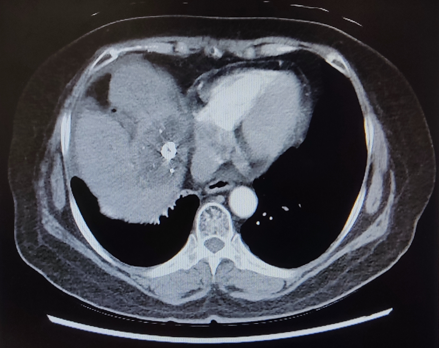** | | |
| --- | --- | --- | --- | --- | --- | --- | --- |
| **A2** | **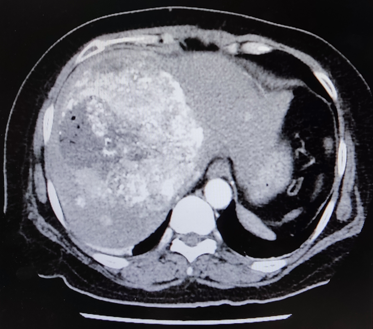** | | | **B2** | **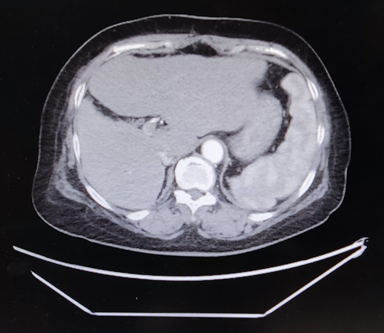** | | |
| **C** | **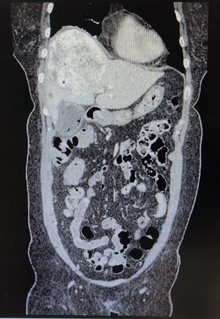** | **D** | **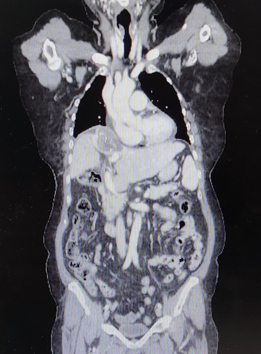** | | | **E** | **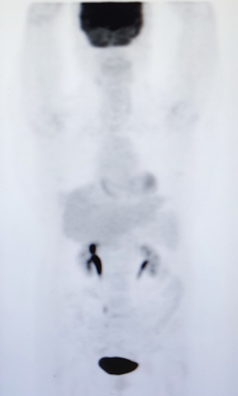** |

1. (1) Arterial phase (2) Axial contrast CT image before SBRT-IO showing bi-lobed HCC
2. (1) Arterial phase (2) Axial contrast CT at 20-month follow-up time after SBRT-IO showing complete response of tumours
3. Coronal contrast CT image before SBRT-IO
4. Coronal contrast CT image at 20-month follow-up time after SBRT-IO
5. Coronal PET/CT image at 20-month follow-up time after SBRT-IO

**Abbreviations:** HCC, hepatocellular carcinoma; SBRT, stereotactic body radiotherapy; CR, complete response; N, number of lesions
